# A systematic review: the current status of carbapenem resistance in East Africa

**DOI:** 10.1186/s13104-018-3738-2

**Published:** 2018-08-31

**Authors:** Kenneth Ssekatawa, Dennis K. Byarugaba, Edward Wampande, Francis Ejobi

**Affiliations:** 1 College of Veterinary Medicine, Animal Resources and Biosecurity, Makerere University, P. O. Box 7062, Kampala, Uganda; 2 Department of Biochemistry, Faculty of Biomedical Sciences, Kampala International University-Western Campus, P. O. Box 71, Bushenyi, Uganda

**Keywords:** East Africa, Molecular epidemiology, Carbapenem resistance

## Abstract

**Objective:**

In this systematic review, we present the molecular epidemiology and knowledge gaps of the carbapenem resistance in East Africa as well as the future probable research interventions that can be used to address the emergence of carbapenem resistance in the region.

**Results:**

The 17 articles which presented concrete information about the prevalence of carbapenem resistance in East Africa were reviewed. Tanzania exhibited the highest level of carbapenem resistance at 35% while DRC had the lowest level at 0.96%. Uganda was the only country with studies documenting CR obtained amongst hospital environment isolates with incidence ranging from 21% in *Pseudomonas aeruginosa* to 55% in *Acinetobacter baumannii.* Carbapenem resistance was more exhibited in *A. baumannii* (23%), followed by *P. aeruginosa* (17%), *Klebsiella pneumoniae* (15%), *Proteus mirabilis* (14%) and *Escherichia coli* (12%) mainly isolated from respiratory tract, blood, urine and wound/pus. The regional genetic determinants of carbapenem resistance detected were *bla*IMP, *bla*VIM-1 *bla*SPM-l, *bla*NDM-1, *bla*OXA-23 *bla*OXA-24, *bla*OXA-58 and *bla*KPC.

**Electronic supplementary material:**

The online version of this article (10.1186/s13104-018-3738-2) contains supplementary material, which is available to authorized users.

## Introduction

In the recent past, carbapenems were potent against all multiple drug resistant (MDR) Gram negative bacteria and in combination with their negligible toxicity to the host, carbapenems became the preferred last resort antibiotics for the treatment of MDR Gram negative bacterial infections. Development of carbapenem resistance (CR) in *Enterobacteriaceae* is of great concern because there is no obvious next line of antibiotics to use against carbapenemase producing (CP) *Enterobacteriaceae* [1]. MDR has left less efficient antibiotics to take care of these expensive hard to treat life threatening infections [2–6].

Currently, the high prevalence of carbapenem resistant *Enterobacteriaceae* (CRE) isolates world over most importantly in *Klebsiella pneumoniae* and *Escherichia coli* isolates in hospitals, community-associated infections and animals is a huge burden to the health care system [3, 5–12], Additional file 2: Table S6. Genetic determinants of CR have been classified into: Ambler class A beta lactamases which include; KPC, GES/IBC, SME, NMC-A, IMI and SFC [12–15], Ambler class B beta lactamases which are termed as Metallo beta Lactamases consisting of NDM, VIM, IMP, SPM, GIM, SIM, KHM, AIM, DIM, SMB, TMB and FIM [7, 13]. IMP, VIM and NDM plasmid mediated Metallo beta lactamases are of worldwide occurrence possibly because the genes that code for them are located on mobile genetic elements [13] and carbapenem hydrolyzing class D beta lactamases (CHDLs) encompass various group of oxacillinases (OXA) with hydrolytic activity of amino and carboxy penicillins [16], Additional file 2: Tables S3–S5.

Studies have reported the existence of CP bacteria in East Africa but in general, there is no comprehensive data about the molecular epidemiology of CP organisms and its burden on the health care system [17, 18]. Furthermore, there is scanty information about CR prevalence in East African livestock yet MDR genes were observed in livestock commensal bacteria which are probably transmitted to humans through the food chain [19–21].

Comprehending the current status of CR throughout East Africa will influence decision making among stakeholders about the rational use of carbapenems. Therefore, this systematic review expounds the current molecular epidemiology of CP bacteria in East Africa, highlighting the carbapenemases genes, CR Knowledge gap and future research interventions to address CR in East Africa.

## Main text

### Methods

#### Literature review

**Fig. 1:**
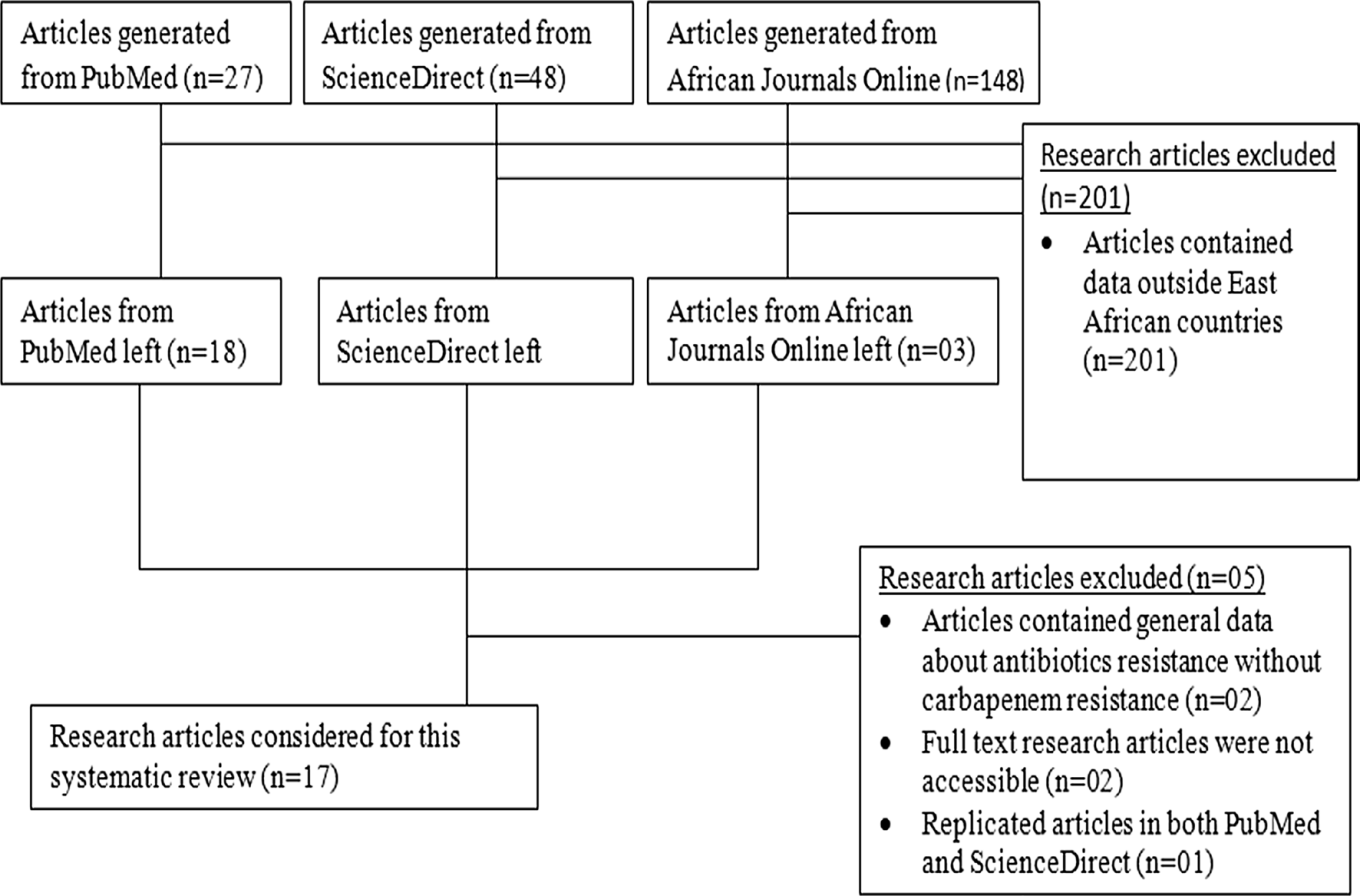
Selection process of research articles for inclusion in this systematic review

PubMed, ScienceDirect and African Journals Online databases were searched from March to December 2017. The search key words used were carbapenem resistance in East Africa to extract articles published only in English from 2008 onwards in an attempt to include up to date relevant CR data, Fig. 1.

#### Study selection criteria

Only full text research articles reporting the prevalence of CP bacteria isolated from patients and hospital environment in East African countries namely; Kenya, Uganda, Tanzania, Burundi, Rwanda, Ethiopia, Democratic Republic of Congo (DRC) and South Sudan were used. Only Studies elaborating bacteria study population, pathogens identified, phenotypic and genotypic methods used to detect CR were used. Patients’ populations of all ages were included while case reports and review articles were excluded from this systematic review as it has become conventional [22].

#### Data extraction

A database was created in which study location, publication year, sample collection period, bacterial species isolated, number of isolates tested for CR, CR prevalence, carbapenemase genes, methods used to identify resistant isolates and to type CR genetic determinants were included, Table 1.

**Table 1:** Review of East Africa based carbapenem resistance studies

Location	Number of isolates	CR isolates	CR prevalence (%)	Carbapenemase genes	Organism	Period	Methods used	Refs
Kenya	416	57	13.7	VIM-2	*P. aeruginosa*	Jan 2006–Jun 2007	PCR, PFGE and sequencing	[23]
Kenya	> 100	7	–	NDM-1	*K. pneumoniae*	2007–2009	PCR, PFGE and sequencing	[24]
Kenya	–	16	–	NDM-1	*A. baumannii*	Jan 2009–Aug 2010	PCR, PFGE, Sequencing and MBL Etest strips	[25]
Kenya	190	44	23.2	–	*K. pneumoniae*	2002–2013	Disk diffusion	[26]
Kenya	195	25	12.8	NDM-1 like	*K. pneumoniae*	1994–2017	WGS	[27]
Kenya	17/219	1/219 or 1/17	0.5/5.9	–	*K. pneumoniae*	2010	Disk diffusion	[28]
Kenya	42	3	7	–	*E. coli*	–	Disk diffusion	[29]
Uganda	196	44	22.4	OXA-48, IMP, KPC and NDM-1	*K. pneumoniae. E. coli, Enterobacter* spp, *Serratia marcescens, Proteus mirabilis, Citrobacter freundii, Klebsiella oxytoca* and *Pantoea agglomerans*	Jan 2013–Mar 2014	PCR. Disk diffusion and Modified Hodge test	[30]
Uganda	658	68	10.3	VIM and OXA-48	*E. coli, K. pneumoniae, Proteus mirabilis, Salmonella* spp, *Morganella morganii, Enterobacter sakazaki* and *Stenotrophomonas* spp	Sept 2013–Jun 2014	PCR and disk diffusion	[31]
Uganda	869 (clinical)	10	1.2 (10/869) 24 (10/42)	IMP-like, VIM-like, SPM-like and NDM-1-like	*P. aeruginosa* (42/658 = 5%)	Feb 2007–Sep 2009	PCR Phoenix Automated Microbiology System	[32]
		9	1.1 (9/869) 31 (9/29)	OXA-23,24, 58 like and VIM-like	*A. baumannii* (29/658 = 3%)			
	80 (environmental)	15	18.8 (15/80) 33 (15/46)	IMP-like, VIM-like, SPM-like and NDM-1-like	*P. aeruginosa* 57.5% (46/80)			
		6	7.5 (6/80) 55 (6/11)	OXA-23,24, 58 like and VIM-like	*A. baumannii* 14% (11/80)			
Uganda	736 (clinical)	3	0.41 (3/736) 33 (3/9)	–	*P. aeruginosa* (9/736 = 1.2%)	Sept 2012–Oct 2013	Rep-PCR and disk diffusion	[33]
		1	0.14 (1/736) 14 (1/7)	–	*A. baumannii* (7/736 = 0.95%)			
	100 (environmental)	7	7 (7/100) 21 (7/33)	–	*P. aeruginosa* (33/100 = 33%)			
		6	6 (6/100) 46 (6/13)	–	*A. baumannii* (13/100 = 13%)			
Tanzania	90	8	8.9	VIM-2	*P. aeruginosa*	May 2010–Jul 2011	Sequencing, PGFE and Disc diffusing	[34]
Tanzania	227	80	35	VIM-, IMP-, NDM-KPC, OXA48	*K. pneumoniae, P. aeruginosa, E. coli, K. oxytoca A. baumannii, Citrobacter freundii, Serratia marcescens* and *Salmonella* spp	2007–2012	PCR and disk diffusion	[35]
Rwanda	55/154	5 in 154 or 5/55	2.9/8	–	*E. coli*	Jul–Dec 2013	Disk diffusion	[36]
Ethiopia	33	4	12.1	–	*K. pneumoniae* and *Morganella morgani*	Jan–Mar 2014	Disc diffusion and Modified Hodge test	[37]
Ethiopia	267	5	1.87	–	*K. pneumoniae* *E. coli*	Dec 2012		[38]
DRC	104/643	1 in 643 or 1/104	0.2/0.96	–	*Enterobacter* spp.	Sept 2012–Aug 2013	Disk diffusion	[39]

#### Data analysis

Data analysis was performed using one-way ANOVA in XLSTAT version 2018.1 to establish the most prevalent carbapenem resistant bacteria type and their distribution variability within body systems. A *P* value of ≤ 0.05 indicated significant statistical difference.

### Results

The search conducted between January and December 2017 generated 223 research articles; PubMed, Sciencedirect and African Journals Online liberated 27, 48 and 148 respectively. Using article abstracts and titles, 201 articles were excluded from this systematic review. Only 20 full text articles were accessible out of the 22 papers. Of the remaining 20 manuscripts, 17 presented concrete information about molecular epidemiology of CR in East Africa and consequently included in this review, Fig. 1. The search generated four manuscripts from Uganda, seven from Kenya, two from Ethiopia, and Tanzania, one from DRC and Rwanda, Table 1. Neither articles from Burundi nor from South Sudan met inclusion criteria for this systematic review. All studies were epidemiological hospital based cross sectional in nature and majority illustrated the prevalence and genetic determinants of CR as well as the methods employed to detect CP isolates, Table 1.

#### Resistance patterns

##### Clinical isolates

According to the molecular and antibiotics susceptibility assays employed in the articles incorporated into this systematic review, Tanzania exhibited the highest level of CR among enteric clinical isolates at 35% while DRC had the lowest level at 0.96% [35, 38], Table 1.

##### Hospital environment isolates

Uganda was the only regional country with two studies documenting CR obtained amongst hospital environment isolates [32, 33]. These studies reported hospital environment CR prevalence ranging from 21% in *P. aeruginosa* to 55% in *A. baumannii,* Table 1.

##### Body system harboring CP isolates

Only 12 articles analyzed in this review had detailed information about the samples from which CP bacteria were isolated, Table 2. The mean sample wise CP bacteria distribution was highest in respiratory tract samples (23%), followed by blood (22%), urine (19%), wound/pus (18%), stool and peritoneol fluid (10%), other samples (7%), ear swabs (6%) and cerebral fluid (3%), Additional file 1: Table S1. Seven studies reported CR in urine and blood isolates with prevalence ranging from 0.96% (DCR) to 39.2% (Tanzania) and 7% (Kenya) to 36.36% (Tanzania) respectively. Six articles documented respiratory tract CP bacteria with occurrence varying from 3.45% (Uganda) to 55.6% (Kenya) while five articles displayed CR in pus/wound isolates with a resistance incidence ranging from 7.14% (Uganda) to 33.04% (Tanzania), Table 2.

**Table 2:** Sample wise distribution of CR bacteria isolates

Country	Sample source	CR prevalence	Species	Refs
Kenya	Urine Blood Wounds Respiratory tract specimens various other specimens	3/57 = 5% (urine) 4/57 = 7% (blood) 17/57 = 30% (wound/pus) 30/47 = 53% (respiratory) 3/57 = 5% (various other specimens)	*P. aeruginosa*	[22]
Kenya	Blood (190)	44/190 = 23.2%	*K. pneumoniae*	[24]
Kenya	Respiratory tract specimens Bone marrow aspirate cerebrospinal fluid Catheter tip Axillary swab Nasal swab Urine Blood Debrided tissue samples	10/16 = 55.6% (respiratory) – – – – – – – –	*A. baumanii*	[25]
Kenya	Blood (195)	25/195 = 12.8%	*K. pneumoniae*	[27]
Kenya	Urine (121)	1/17 = 5.9%	*K. pneumoniae*	[28]
Uganda	Urine Blood Stool Wound/pus Peritoneol fluid Others	23% 27% 18% 14% 10% 8%	*E. coli, K. pneumoniae, Proteus mirabilis, Salmonella* spp, *Morganella morganii, Enterobacter sakazaki* and *Stenotrophomonas* spp	[31]
Uganda	Blood (51), cerebral spinal fluid (49), Tracheal aspirates (163), Ear swabs (197), Sputum (204), Urine catheters (98) and Pus (107)	3/42 = 7.14% (wound/Pus) 3/42 = 7.14% (Sputum) 3/42 = 7.14% (Tracheal) 1/42 = 2.4% (Ear swab)	*P. aeruginose*	[32]
		4/29 = 13.8% (Tracheal) 3/29 = 10.35% (Ear swap) 1/29 = 3.45% (Sputum) 1/29 = 3.45% (Cerebral Spinal fluid)	*A. baumanii*	
Tanzania	Blood and pus	5/90 = 5.6% Wound/Pus 3/90 = 3.3% Blood	*P. aeruginose*	[34]
Tanzania	Pus (112), urine (56), blood (55), aspirate (3), and sputum (1).	22/56 = 39.29% (Urine) 20/55 = 36.36% (Blood) 37/112 = 33.04% (wound/Pus)	*K. pneumoniae, P. aeruginosa, E. coli, K. oxytoca A. baumannii, Citrobacter* *freundii, Serratia marcescens* and *Salmonella* spp	[35]
Ethiopia	Urine (24) Blood (9)	4/24 = 17% (urine) 0/9 = 0% (blood)	*K. pneumoniae* and *Morganella morgani*	[37]
Ethiopia	Feces (267)	5/267 = 2% (feces)	*K. pneumoniae* *E. coli*	[38]
DRC	Urine (104)	1/104 = 0.96% (urine)	*Enterobacter* spp	[39]

##### Distribution of CR among MDR enteric bacteria

One-way ANOVA displayed that distribution of CR among different bacteria species was not significantly different (P-value = 0.11 > 0.05). CR prevalence was highest in *A. baumannii* with an average of 23% followed by *P. aeruginosa* (17*%*), *K. pneumonia* (15%), *P. mirabili* (14%), *E. coli* (12%), *C. freundii* (8%), *K. oxytoca* (2%), *M. morganii* (2%), *Salmonella* spp *E. sakazaki* and *Stenotrophomonas* spp (1%). However, the most reported CP isolate across the region was *K. pneumoniae* (8 studies) followed by *E. coli* and *P. aeruginosa* (6 studies), *A. baumannii* (4 articles), *M. morganii* and *Salmonella* spp (2 articles)*, C. freundii*, *K. oxytoca* and *P. mirabilis* (2 articles), *E. sakazaki* and *Stenotrophomonas* spp (1 article), Additional file 1: Table S2.

#### Prevalence of CR genetic determinants in East Africa

##### Uganda

CR genetic determinants in non-glucose fermenting bacteria reported at Mulago hospital were *bla*IMP-like (36%), *bla*VIM-like (32%), *bla*SPM-like (16%), *bla*NDM-1-like (4%) for *P. aeruginosa* and *bla*OXA-23-like (60%), *bla*OXA-24-like (7%), *bla*OXA-58-like (13%), and *bla*VIM-like (13%) for *A. baumannii* [32]. Carbapenemase genes in CRE at Mulago and Mbarara hospitals were also documented [30, 31]. At Mulago, the genes characterized included; blaVIM (10.7%), followed by blaOXA-48 (9.7%), blaIMP (6.1%), blaKPC (5.1%) and blaNDM-1 (2.6%). The highest number of genes appeared in *Klebsiella pneumoniae* (52.2%), followed by *E. coli* (28.4%), *Enterobacter* spp (7.5%), *Serratia marcescens* (4.5%), *Proteus mirabilis* (3.0%), *Citrobacter freundii, Klebsiella oxytoca*, and *Pantoea agglomerans* at 1.5% each while at Mbarara hospital, VIM and OXA-48 CR determinants were registered, Table 1.

##### Tanzania

Molecular analysis of CRE at a tertiary hospital in Mwanza established by multiplex PCR revealed that the principal CR genes were IMP (21.6%), followed by VIM (12.3%), OXA-48 (4.9%), then KPC (3.5%), and NDM (3.1%). CP *E. coli* had the highest prevalence (14%), followed by *K. pneumoniae* (10.57%), trailed by *P. aeruginosa* (10.13%), then *Klebsiella oxytoca* (1.76%), *A. baumannii* (1.3%), *C. freundii* (0.88%), *Serratia marcescens* (0.88%) and *Salmonella* spp. (0.44%) [35] while CP *P. aeruginosa* harbouring VIM CR gene were identified from Muhimbili National Hospital, using PCR [34], Table 1.

##### Kenya

CP *K. pneumoniae*, *A. baumannii* and *Pseudomonas aeruginosa* possessing NDM and VIM-2 genes respectively were isolated in Nairobi [21, 24, 25] while Whole Genome sequencing (WGS) was employed to identify NDM-1like CR genes in *K. pneumoniae* isolates at Kilifi County Hospital [27], Table 1.

### Discussion

#### Geographical prevalence of CP bacteria

The most prevalent CRE across the region were *K. pneumoniae* and *E. coli*. CR in *K. pneumoniae* was reported by eight articles with mean prevalence of 15% in all East African countries except Rwanda and DRC while CP *E. coli* was accounted for in all countries apart from DCR by six studies with an average occurrence of 12%. This is in agreement with global data about CR. For example in USA, 11% of *K. pneumoniae* infections and 2% of *E. coli* infections were resistant to carbapenems [40] while in India, 13% of *E. coli* infections and 57% of *K. pneumonia* infections were caused by CP strains [41]. Additionally, high frequency of CR among the non-glucose fermenting *P. aeruginosa* (17%) and *A. baumannii* (23%) almost equal to that of CRE was registered in the region (Table 1 and Additional file 1: Table S2). This is in conformity with worldwide reports acknowledging that the magnitude of CP *A. baumannii* and *P. aeruginosa* is equal to that of CRE [40].

#### Prevalence of CR

The highest frequency of CR in the region was 35%. This prevalence correlates with other studies in India [43, 44] where the prevalence was 43% and 30% respectively. Contrary, this frequency is higher than CR levels reported by other studies in Nigeria (15.2%) and USA (4.5%) but lower than that of 68% reported by a broad study executed in 7 out of the 9 provinces of South Africa [45–47].

#### Carbapenem resistant bacteria in the hospital environment

The actual occurrence of environmental contamination by CP bacteria is not well researched yet hospital environments tainted with CP bacteria by infected patients are implicated as the main routes of transmission [48, 49]. Across East Africa, only two studies conducted in Uganda reported the existence of CP *P. aeruginosa* and *A. baumannii* isolated from hospital environment. The frequency varied from 21% in *P. aeruginosa* to 55% in *A. baumannii,* Table 1. Related studies which recovered CP bacteria from hospital environments in Israel and Brazil reported closely related results [50–52]. Horizontal transfer of mobile genetic elements from clinical pathogens to environmental bacteria can occur within the hospital environment hence promoting emergence of new resistant bacteria strains. Furthermore, resistant bacteria in hospital environment such as sewage may spill into the food chain, hence becoming one of the sources of community-acquired resistant pathogens [40].

#### Sample wise distribution of CP bacteria

This systematic review revealed that CP bacteria are highly distributed in the respiratory tract (23%), Blood (22%), urinary tract (19%) and wounds/pus (18%) in East Africa and this is in line with other investigations conducted in India [44, 53] and USA [46], where they reported high incidences of CR respiratory tract, urinary tract, blood and wound bacterial infections.

#### CR knowledge gap in East Africa

Various studies around the global have characterized the different variants of each genetic determinant of CR, Additional file 2: Tables S3–S5. Unfortunately, all these variants and their epidemiology are yet to be documented in East Africa, S-CRKGEA. Emergence of CR in *K. pneumonia* strains harbouring Extended Spectrum Beta-Lactamases-ESBLS (CTX-Ms or SHV-2) or plasmid borne AmpC enzymes (ACT-1, CMY-2, CMY-4 or DHA-1) in association to loss of outer membrane proteins (OMPs) as a result of truncated OMP gene [54, 55] is yet to be acknowledged in East Africa. Occurrence of CP bacteria in livestock and their environment was reported in Europe [9–12] while in East Africa no such research has ever been conducted.

## Conclusion and recommendation

Identification of CP bacterial infections at their first appearance provides an opportunity to interfere before these CP organisms are spread more extensively [6]. Therefore, utilizing a robust molecular platform, the WGS, all genetic determinants of CR in humans, livestock and environment should be identified and documented hence bridging the knowledge gap about the molecular epidemiology of CP bacteria in East Africa. Antibiotics resistance stewardship team may profit from data generated by molecular testing of MDR organisms to enhance prevention of intra and inter health facility transmission and the possible cyclic transmission between livestock, humans and environment.

## Limitations

Results of the 17 articles that illustrated significant CR across East Africa have been summarized by this review. However, in South Sudan and Burundi no studies have ever been conducted to investigate the epidemiology of CR. Furthermore, studies carried out in Rwanda, DRC, and Ethiopia were aimed at addressing general antimicrobial resistance hence providing very limited CR data while in Kenya, Uganda and Tanzania, more elaborate specific CR in enteric bacteria studies have be performed. Therefore this has led to a significant variation in knowledge about CR in the region.

## Additional files

**Additional file 1.** One way ANOVA results. **Table S1.** Mean percentage of sample wise distribution of carbapenem resistant isolates generated by One-Way ANOVA. **Table S2.** Average percentage prevalence of the different carbapenem resistant bacteria computed by One-Way ANOVA.

**Additional file 2.** Class A, Class B and Class D genetic determinants of Carbapenem resistance. a. **Table S3**. Showing Ambler class A carbapenemase, their variants, organisms harbouring them and location. b. **Table S4.** MBLs, their variants, organisms harbouring them and location. c. **Table S5.** Carbapenem-hydrolyzing class D β-lactamases (CHDL), organisms harbouring them, their geographic distribution and location. d. **Table S6.** Shows the prevalence of carbapenem resistance in K. pneumonia in all WHO regions. e. Carbapenem resistance Knowledge gap in East Africa (S-CRKGEA).

## Abbreviations

ACT: AmpC type
AIM: Australian imipenemase
AmpC: amino penicillin cephalosporinase
ANOVA: analysis of variance
bla: beta lactamase
CHDL: carbapenem hydrolyzing class D beta lactamase
CMY: cephamycins
CP: carbapenemase producing
CR: carbapenem resistance
CRE: carbapenem resistant *Enterobacteriaceae*
CTX: cephotaxime hydrolyzing capabilities
DHA: Dhaharani Hospital
DIM: Dutch imipenemase
DRC: Democratic Republic of Congo
ESBL: extended spectrum beta-lactamases
FIM: florence imipenemase
GES: Guiana extended Spectrum enzyme
GIM: German imipenemase
IBC: integron-borne cephalosporinase
IMI: imipenem hydrolyzing lactamase
IMP: imipenemase Metallo beta lactamase
KHM: Kyorin University Hospital
KPC: *Klebsiella pneumoniae* carbapenemase
MBL: Metallo beta lactamase
MDR: multi-drug resistant
NDM: New Delhi Metallo beta lactamase
NMC: not metalloenzyme carbapenemase
OMP: outer membrane protein
OXA: oxacillinases
Ref: reference
SFC: *Serratia fonticola* carbapenemase
SHV: sulfhydryl variables
SIM: Seoul imipenemase
SMB: *S. marcescens* Metallo beta lactamase
SME: *Serratia marcescens* enzyme
SPM: Sao Paulo Metallo beta lactamase
TMB: Tripoli Metallo beta lactamase
VIM: Verona Integron encoded Metallo beta lactamase
