# Supplementary material for: A systematic review: the current status of carbapenem resistance in East Africa
Source: BMC Res Notes. 2018 Aug 31;11:629. doi: 10.1186/s13104-018-3738-2 (PMC6119249; doi:10.1186/s13104-018-3738-2)
Supplement: Supplementary file 1 — Additional file 1. One way ANOVA results. Table S1. Mean percentage of sample wise distribution of carbapenem resistant isolates generated by One-Way ANOVA. Table S2. Average percentage prevalence of the different carbapenem resistant bacteria computed by One-Way ANOVA. [file 13104_2018_3738_MOESM1_ESM.docx]

| **Sample type** | **No of studies** | ***Sum of Percentage of Carbapenem resistant isolates*** | ***Average percentage of carbapenem resistant isolates*** | ***Variance*** |
| --- | --- | --- | --- | --- |
| Urine | 7 | 135% | 19% | 0.029423118 |
| blood | 7 | 154% | 22% | 0.023231006 |
| Wound/pus | 5 | 90% | 18% | 0.016447288 |
| Respiratory tract infections | 6 | 140% | 23% | 0.058638511 |
| Stool | 2 | 20% | 10% | 0.0128 |
| Ear swabs | 2 | 13% | 6% | 0.003160125 |
| Cerebral fluid | 1 | 3% | 3% | - |
| Peritoneol fluid | 1 | 10% | 10% | - |
| others | 2 | 13% | 7% | 0.00045 |

# *Table S1: Mean percentage of sample wise distribution of carbapenem resistant isolates generated by One-Way ANOVA*

P-value =0.82 > 0.05 indicating that there is no significant difference in body systems distribution of carbapenem resistant isolates

## *Table S2: Average percentage prevalence of the different carbapenem resistant bacteria computed by One-Way ANOVA*

| ***Bacteria type*** | ***NO of studies*** | ***Sum of percentage of CR prevalence*** | ***Average percentage prevalence CP bacteria*** | ***Variance*** |
| --- | --- | --- | --- | --- |
| *K. pneumoniae* | 8 | 119% | 15% | 0.009732 |
| *E.coli* | 6 | 73% | 12% | 0.010943 |
| *P. aeruginosa* | 6 | 103% | 17% | 0.006961 |
| *A. baumannii* | 4 | 91% | 23% | 0.026888 |
| *C. freundii* | 2 | 16% | 8% | 0.003715 |
| *P. mirabilis* | 2 | 28% | 14% | 0.032411 |
| *K. oxytoca* | 2 | 5% | 2% | 0.001176 |
| *P. agglomerans* | 1 | 0% | 0% | - |
| *Salmonella spp* | 3 | 4% | 1% | 0.00012 |
| *M. morganii* | 3 | 5% | 2% | 0.00017 |
| *E. sakazaki* | 1 | 1% | 1% | - |
| *Stenotrophomonas spp* | 1 | 1% | 1% | - |

P-value =0.11 > 0.05 indicating that there is no significant difference in the prevalence of the different types of carbapenem resistant bacteria
