# Supplementary material for: A systematic review: the current status of carbapenem resistance in East Africa
Source: BMC Res Notes. 2018 Aug 31;11:629. doi: 10.1186/s13104-018-3738-2 (PMC6119249; doi:10.1186/s13104-018-3738-2)
Supplement: Supplementary file 2 — Additional file 2. Class A, Class B and Class D genetic determinants of Carbapenem resistance. a. Table S3. Showing Ambler class A carbapenemase, their variants, organisms harbouring them and location. b. Table S4. MBLs, their variants, organisms harbouring them and location. c. Table S5. Carbapenem-hydrolyzing class D β-lactamases (CHDL), organisms harbouring them, their geographic distribution and location. d. Table S6. Shows the prevalence of carbapenem resistance in K. pneumonia in all WHO regions. e. Carbapenem resistance Knowledge gap in East Africa (S-CRKGEA). [file 13104_2018_3738_MOESM2_ESM.doc]

### Carbapenemase encoding genes in *Enterobacteriaceae* and other enteric bacteria

Acquisition of carbapenemase genes which encode for enzymes with potency of degrading carbapenems, reduced absorption of antibiotics by a qualitative and quantitative deficiency of porin expression in conjunction with over expression of beta-lactamases which exhibit very fragile affinity for carbapenems and modification in the affinity of the target enzymes, the penicillin binding proteins for carbapenems have been illustrated as the major mechanisms of carbapenem resistance in *Enterobacteriaceae* and other enteric bacteria [1-2]. These carbapenemases are classified as; Molecular class A serine carbapenemases of functional group 2f, Class B Metallo-Lactamases and Class D serine-carbapenemases or the OXA-beta lactamases.

**Ambler class A carbapenemases**

Many Ambler class A beta lactamases have been identified but only some have demonstrated carbapenem hydrolyzing activity [3].Presence of an active site serine at position 70 in the Ambler numbering system is a prerequisite for the hydrolytic activity of class A beta lactamases [4]Those with carbapenem hydrolysis activity include; *Klebsiella pneumoniae* Carbapenemase (KPC_2-12_) and Guiana extended Spectrum enzyme / integron-borne cephalosporinase (GES_2,4,5,6,11&14_/IBC) encoded for by KPC and GES/IBC respectively which are plasmid harbored genes as well as *Serratia marcescens* enzyme (SME_1-3_), Not Metalloenzyme carbapenemase (NMC-A ), imipenem hydrolyzing lactamase (IMI_1-2_) *Serratia fonticola* carbapenemase (SFC_1_),and BIC beta lactamases , coded for by SME, NMCa IMI, SFC and BIC in that order which are chromosomally located genes [1-2] as shown by Table S3 adopted from[5]. All ambler class A beta lactamases possess the capacity to hydrolyze a broad range of other beta lactams, including cephalosporins, penicillins, and aztreonam except for GES which has no hydrolytic activity for aztreonam, and all are inhibited by the presence clavulanate and tazobactam [1]Of all molecular class A serine carbapenemases, KPC and GES are worth the attention of clinician due to the fact that currently, their prevalence is universal [3]

**Ambler class B carbapenemases: Metallo-**β**-lactamases (MBLs)**

Ambler class B beta lactamases are termed as Metallo Beta Lactamases and hence need a metal ion, in most cases zinc for beta lactam hydrolysis [6]. Presence of metal chelating agents like EDTA inhibits hydrolysis of beta lactam antibiotics by MBLs because of their dependence on Zinc ion [6].Metallo beta lactamases secretion in gram negative bacteria presents resistance to all beta lactam antibiotics including the last generation carbapenems but short of ability to break down aztreonam and are not inhibited by the presence of beta lactamase inhibitors clavulanate and tazobactam[1,3]. Class B Metallo Beta Lactamases which consist of, New Delhi Metallo beta lactamase (NDM_1-6_), Verona Integron-encoded Metallo bata lactamase (VIM_1-33_), Imipenemase Metallo beta lactamase (IMP_1-33_), Sao Paulo Metallo beta lactamase (SPM_1_), German imipenemase (GIM_1_), Seoul imipenemase (SIM_1_), Kyorin University Hospital (KHM_1_), Australian imipenemase (AIM_1_), Dutch imipenemase (DIM-_1_), *S. marcescens* MBL (SMB-_1_), Tripoli MBL (TMB-_1_)) and Florence imipenemase (FIM-_1_) [3]Table S4. IMP, VIM and NDM plasmid mediated metallo beta lactamases are of worldwide occurrence possibly because the genes that code for them are located on mobile genetic elements [3]. While SPM_1_ isolated in Sao Paulo-Brazil [7-8]; GIM isolated in Germany [9]; SIM identified in Seoul Korea (Lee *et al.,* 2005); KHM identified from Kyorin Health Science*-*Japan [10]; AIM discovered in Australia [11]; DIM isolated in Netherlands [12]; SMB first revealed in Japan [13]; TMB isolated in Libya [14], and FIM classified in Italy [15], have remained restricted to the countries they were first isolated [16].

**Class D serine-carbapenemases (OXA-beta lactamases)**

Carbapenem hydrolyzing class D beta lactamases encompass various group of oxacillinases with hydrolytic activity of amino and carboxy penicillins [17]. A big group of oxacillinases is not inhibited by EDTA and clavulanate but inhibited by NaCl *in vitro* [3]. Different researchers have reported over 250 types of oxacillinases but very few have the potency of carbapenem degradation. Table S5 shows the families of oxacillinases with carbapenem hydroltytic activity also termed as the carbapenem hydrolyzing class D beta lactamase (CHDL), their host organisms and geographic distribution [3]. High prevalence of CHDLs has been reported in *Acinetobacter* spp but also an increasing occurrence of CHDLs in particular OXA-48 and 181 has been observed in *Enterobacteriaceae* [1,18-19]. Penicillins, first generation cephalosporins, and beta lactamase inhibitor combinations are susceptible to CHDLs while extended spectrum cephalosporins are not. The efficacy of carbapenem hydrolysis by CHDLs is less than that of other carbapenemases. Therefore, in most cases, a combination CHDLs and other resistance mechanism such as production of other carbapenemases, modification in outer membrane proteins [20-22], amplified transcription mediated by *IS* elements performing as promoters, amplified gene copy number and increased drug efflux [20,23] has been employed in organisms exhibiting elevated levels of phenotypic carbapenem resistance.

## Table S3: Showing Ambler class A carbapenemase, their variants, organisms habouring them and location

| **Enzyme** | **Variants** | **Organism (s)** | **Gene location** | **Reference** |
| --- | --- | --- | --- | --- |
| KPC | KPC-2 | *Enterobacteriaceae, P.aeruginosa, Acinetobacter spp*. | Plasmid | [24] |
|  | KPC-3 | *Enterobacteriaceae, Acinetobacter spp* | plasmid | [25] |
|  | KPC-4 | *Enterobacter cancerogenus, K.pneumoniae, Acinetobacter spp.* | plasmid | 26-27] |
|  | KPC-5 | *P. aeruginosa* | plasmid | [28] |
|  | KPC-6,7,8 &11 | *K. pneumoniae* | Plasmid & unknown for KPC11 | [3,27,29-31] |
|  | KPC-9 &12 | *E. coli* | Plasmid & unknown for KPC12 | [3,32] |
|  | KPC-10 | *Acinetobacter* spp. | plasmid | [27] |
|  | KPC-13 | *Enterobacter cloacae* | unknown | [3] |
| GES | GES-2 | *P. aeruginosa* | Plasmid | [33] |
|  | GES-4&6 | *K. pneumoniae* | Plasmid | [34-35] |
|  | GES-5 | *K. pneumoniae*, *E. coli*, *P.aeruginosa* | Plasmid | [35-36] |
|  | GES-11&14 | *Acinetobacter baumannii* | Plasmid | [37-38] |
| SME | SME-1,2 &3 | *S. marcescens* | chromosomal | [39-43] |
| NmcA |  | *Enterobacter cloacae* | chromosomal | [44] |
| IMI | IMI-1 | *Enterobacter cloacae* | chromosomal | [45] |
|  | IMI-2 | *Enterobacterasburiae*, *Enterobacter cloacae* | Plasmid | [46-47] |
| SFC | SFC-1 | *S. fonticola* | Chromosomal | [48] |
| BIC | BIC-1 | *P. fluorescens* | Chromosomal | [49] |

## Table S4: MBLs, their variants, organisms habouring them and location

| **Enzyme** | **Variants** | **Organism (s)** | **Gene location** | **Reference** |
| --- | --- | --- | --- | --- |
| IMP | IMP-1 to 42 | Enterobacteriaceae, *Pseudomonas* spp., *Acinetobacter* spp. | Plasmid or chromosome | [50-51] |
| VIM | VIM-1 to 37 | Enterobacteriaceae, *Pseudomonas* spp., *Acinetobacter* spp. | Plasmid or chromosome | [52-53] |
| NDM | NDM-1 to 7 | Enterobacteriaceae, *Acinetobacter* spp., *Vibriocholerae* | Plasmid or chromosome | [54-56 |
| SPM | SPM-1 | *P. aeruginosa* | Chromosome | [7] |
| GIM | GIM-1 | *P. aeruginosa* | Plasmid | [9] |
| SIM | SIM-1 | *A. baumannii* | Chromosome | [57] |
| AIM | AIM-1 | *P.aeruginosa* | Chromosome | [11]) |
| KHM | KHM-1 | *C. freundii* | Plasmid | [10] |
| DIM | DIM-1 | *P.stutzeri* | Plasmid | [12] |
| SMB | SMB-1 | *S.marcescens* | Chromosome | [13] |
| TMB | TMB-1 | *Achromobacter xylosoxidans* | Chromosome | [14] |
| FIM | FIM-1 | *P.aeruginosa* | Chromosome | [15] |

## Table S5: Carbapenem-hydrolyzing class D β-lactamases (CHDL), organisms habouring them, their geographic distribution and location

| **CHDL group** | **Organism (s)** | **Geographic distribution** | **Gene location** | **Reference** |
| --- | --- | --- | --- | --- |
| OXA-23/27 | *Acinetobacterbaumannii*, *Proteusmirabilis* | Europe, USA, Middle East, Asia, Australia | Plasmid and Chromosomal | [58-59] |
| OXA-24/40 | *A. baumannii* | Europe and USA | Plasmid and Chromosomal | [60-61] |
| 0XA-25 | *A. baumannii* | Spain | Chromosomal | [58] |
| OXA-26 | *A. baumannii* | Belgium | Chromosomal | [58] |
| OXA-48 | *K. pneumoniae*, Enterobacteriaceae | Turkey, Middle East, Northern Africa, Europe, India, USA | Plasmid | [62] |
| OXA-51/66/69 | *A. baumannii* | Worldwide | Chromosomal | [63-64] |
| OXA-58 | *A. baumannii* | Europe, USA, Middle East, South America | Plasmid | [65] |
| OXA-143 | *A. baumannii* | Brazil | Plasmid | [66] |
| OXA-162 | Enterobacteriaceae | Germany | Plasmid | [22] |
| OXA-163 | *K. pneumoniae*, *E. coli* | Argentina and Egypt | Plasmid | [67][68] |
| 0XA-181 | *K. pneumoniae*, *E. coli* | India | Plasmid | [69] |
| OXA-204 | *K. pneumoniae* | Tunisia | Plasmid | [70] |
| 0XA 232 | *K. pneumoniae* | France | Plasmid | [71] |

# *Table S6: Shows the prevalence of carbapenem resistance in K. pneumonia in all WHO regions*

| **Region** | **No of countries** | **Type of data** | **Overall reported**  **range of resistant**  **Proportion (%)** | **Reported range of resistant Proportion (%) in invasive isolates** |
| --- | --- | --- | --- | --- |
| Africa | 4 | National data | 0-4 | - |
| America | 17 | National data and Report to ReLAVRA | 0-11 | - |
|  | 2 | Publications (2) | 0-2 | - |
| Eastern Mediterranean | 4 | National data | 0-54 | 54 |
|  | 1 | Surveillance network | 6 | - |
|  | 5 | Publications (9) | 0-21 | 0 |
| Europe | 31 | National data/report to EARS-Net | 0-68 | 0-68 |
|  | 2 | Publications (3) | 2-7 | 2 |
| South-East Asia | 4 | National data | 0-8 | - |
|  | 2 | Publications (15) | 0-55 | 0-52 |
| Western Pacific | 9 | National data | 0–8 | - |
|  | 1 | Institute surveillance | 0–1 | - |
|  | 2 | Publications (2) | 0–11 | - |

## EARS-Net, European Antimicrobial Resistance Surveillance Network; PAHO, Pan American Health Organization; ReLAVRA, Latin American Antimicrobial Resistance Surveillance Network.

**Carbapenem resistance Knowledge gap in East Africa (S-CRKGEA)**

Various studies around the global have characterized the different variants of each genetic determinant of carbapenem resistance in *Enterobacteriaceae* and other enteric bacteria. Presently, KPC has 12 subtypes-KPC_2-13_ [28,72-75], GES has various variants with GES_2, 11_ and _14_ confined to *Pseudomonaceae* and *Acinetobacter* while GES_4,5_ and _6_ were detected in *Enterobacteriaceae*, SME has three variants-SME_1-3_, NDM has seven alternatives NDM_1-6_ with NDM_1_ the commonest , VIM exists in thirty three forms-VIM_1-33_, and the same applies to IMP-IMP_1-33_ [76]and all these variants and their epidemiology are yet to be documented in East and South Africa. Currently, there is no data about the epidemiology of NMC-A and IMI_2_ which are chromosomally located carbapenemase producing genes in East and South Africa. Furthermore, SPM, GIM, SIM and KHM_1_ [3]carbapenemase producing genes prevalence remains uncertain in East Africa [77].

Class D serine-carbapenemases**/**the OXA-beta lactamases with carbapenemase activity comprising of thirteen variants of Oxallin hydrolyzing (OXA) are well documented in Europe and the US [1-3]. Beta-lactamases OXA_23, 24,48,51_ and _58_ were identified in East Africa [78-81] while there is no information about the epidemiology of other OXA-beta lactamases variants-OXA_25, 16, 143, 162, 163, 181, 204_ and _232_.

Emergence of carbapenem resistance in *K. pneumonia* strains harboring Extended Spectrum Beta-Lactamases-ESBLS (CTX-Ms or SHV-2) or plasmid-borne AmpC enzymes (ACT-1, CMY-2, CMY-4 or DHA-1) associated to loss of outer membrane proteins (OMPs), including OmpK35 and OmpK36 as a result of truncated OMP gene [82-83] is yet to be acknowledged in East Africa.

**References**

1. Queenan A. M and Bush K. (2007). Carbapenemases: the Versatile Lactamases. Johnson & Johnson Pharmaceutical Research & Development, L.L.C., Raritan, New Jersey *08869* *American Society for Microbiology,* 20 (23), 440–458
2. CDC. (2015). Facility Guidance for Control of Carbapenem-resistant *Enterobacteriaceae* (CRE)
3. Patel G. & Bonomo RA. (2013). Stormy waters ahead”: global emergence of carbapenemases. *Frontiers in Microbiology;* doi: 10.3389/fmicb.2013.00048
4. Ambler RP, Coulson AFW, Frere JM, Ghuysen JM, Joris B, Forsman M, Levesque RC, Tiraby G. & Waley SG. (1991). A standard numbering scheme for the class A Beta lactamases. *Biochem. J;* 276:269–270.
5. Walther-Rasmussen J. & Høiby N. (2007). Class A carbapenemases. *J. Antimicrob. Chemother*; 60:470–482.
6. Walsh TR, Weeks J, Livermore DM. & Toleman MA. **(**2011). Dissemination of NDM-1 positive bacteria in the New Delhi environment and its implications for human health: an environmental point prevalence study. Lancet Infect. Dis. **11**:355–362.
7. Toleman MA. &Walsh TR. (2011). Combinatorial events of insertion sequences and ICE in Gram-negative bacteria. *FEMS Microbiol. Rev;* 35:912–935
8. Rossi F. (2011). The challenges of antimicrobial resistance in Brazil. *Clin. Infect.Dis.* 52, 1138–1143.
9. Castanheira M, Toleman MA, Jones RN, Schmidt FJ. & Walsh TR. (2004b). Molecular characterization of a beta-lactamase gene, blaGIM-1 , encoding a new subclass of metallo-beta-lactamase. Antimicrob Agents Chemo ther 48:4654–4661. <https://doi.org/10.1128/AAC.48.12.4654-4661.2004>.
10. Sekiguchi J, Morita K, Kitao T. & Watanabe N, *et al.* (2008). KHM-1, a novel plasmid-mediated metallo-blactamase from a Citrobacter freundii clinical isolate. *Antimicrob. Agents Chemother*. 52, 4194–4197
11. Yong D, Walsh TR, Bell J, Ritchie B, Pratt R. & Toleman MA. (2007). “A novel subgroup metallo β-lactamase, AIM-1, emerges in *Pseudomonas aeruginosa* from Australia abstr.C1-593,”in *47th Annual Inter science Conference on Antimicrobial Agents and Chemotherapy* (Washington DC, American Society for Microbiology).
12. Poirel L, Rodríguez-Martínez J-M, Naiemi NA, Debets-Ossenkopp YJ. & Nordmann P. (2010c). Characterization of DIM-1, an Integron-Encoded Metallo Lactamase from a *Pseudomonas stutzeri* Clinical Isolate in the Netherlands. *Antimicrobial Agents and Chemotherapy;* 54:420–2424
13. Wachino J, Yoshida H, Yamane K, Suzuki S, Matsui M, Ya­magishi T, Tsutsui A, Konda T, Shibayama K. & Arakawa Y. (2011). SMB-1, a novel subclass B3 metallo-beta-lactamase, asso­ciated with IS*CR1* and a class 1 integron, from a carbapen­em-resistant *Serratia marcescens* clinical isolate. *Antimi­crob Agents Chemother;*55:5143-9.
14. El Salabi A, Borra PS, Toleman MA, Samuelsen Ø. & Walsh TR. (2012). Genetic and biochemical characterization of a novel metallo-beta-lactamase, TMB-1, from an *Achromobacter xylosoxidans* strain isolated in Tripoli, Libya. Antimicrob *Agents Chemother;* 56:2241-5.
15. Pollini S, Maradei S, Pecile P, Olivo G, Luzzaro F, Docquier JD. & Rossolini GM. (2013). FIM-1, a new acquired metallo-beta-lactamase from a Pseudomonas aeruginosa clinical isolate from Italy. *Antimicrob Agents Chemother;*57:410-6.
16. Salabi AE, Toleman MA, Weeks J, Bruderer T, Frei R. & Walsh TR. (2010). First report of the metallo-beta-lactamase SPM-1 in Europe. *Antimicrob Agents Chemother* ;54:582
17. Poirel L, Naas T. & Nordmann P. (2010b). Diversity, epidemiology, and genetics of class D -lactamases. *Antimicrob. Agents Chemother*; 54:24–38.
18. Lascols C, Peirano G, Hackel M, Laupland KB. & Pitout JD. (2012). Surveillance and molecular epidemiologyof *Klebsiellapneumoniae* that produce carbapenemases; the first report of OXA-48-like enzymes in NorthAmerica. *Antimi- crobAgentsChemother.* 57, 130–136. Lauretti,L.,Riccio,M.L.,Mazzariol.
19. Mathers AJ, Hazen KC, Carroll J, Yeh AJ, Cox HL, Bonomo RA, *et al.(*2012). First clinical cases of OXA-48 producing carbapenem resistant *Klebsiella pneumoniae* in the United States: the“menace”arrives in the New World. *J. Clin.Microbiol;* 51:680–683.
20. Perez F, Hujer AM, Hujer KM, Decker BK, Rather PN. & Bonomo RA. (2007). Global challenge of multidrug-resistant *Acinetobacter baumannii*. *Antimicrob.Agents Chemother;* 51:3471–3484.
21. Gulmez D, Woodford N, Palepou MF, Mushtaq S, Metan G, Yakupogullari Y, Kocagoz S, Uzun O, Hascelik G.& Livermore DM. (2008). Carbapenem-resistant Escherichia coli and Klebsiella pneumoniae isolates from Turkey with OXA-48-like carbapenemases and outer membrane protein loss. *Int. J. Antimicrob. Agents;* 31: 523–526.
22. Pfeifer Y, Schlatterer K, Engelmann E, Schiller RA, Frangenberg HR, Stiewe D., *et al*. (2012) .Emergence of OXA-48 type carbapenemase producing Enterobacteriaceae in German hospitals. *Antimicrob.Agents Chemother;* 56:2125–2128.
23. Poirel L. & Nordmann P. (2006). Genetic Structures at the Origin of Acquisition and Expression of the Carbapenem-Hydrolyzing Oxacillinase Gene *bla*_OXA-58_ in *Acinetobacter baumannii*. Antimicrobial Agents and Chemotherapy; 50:1442–1448
24. Yigit H, Queenan AM, Anderson GJ, Domenech-Sanchez A, Biddle JW, Steward CD, Alberti, S, Bush K. & Tenover FC. (2001). Novel carbapenem-hydrolyzing beta-lactamase, KPC-1, from a carbapenem-resistant strain of Klebsiella pneumoniae. *Antimicrob. Agents Chemother*; 45:1151–1161.
25. Woodford N, Ward ME, Kaufmann ME, Turton J, Fagan EJ, James D, Johnson AP, Pike R, Warner M, Cheasty T, Pearson A, Harry S, Leach JB, Loughrey, A., Lowes JA, Warren RE. & Livermore DM. (2004). Community and hospital spread of Escherichia coli producing CTX-M extended-spectrum beta-lactamases in the UK. J. *Antimicrob. Chemother;* 54:735–743.
26. Palepou MF, Woodford N, Hope R, Colman M, Glover J, Kaufmann M, *et al.* (eds).(2005).“Novel class A carbapenemase, KPC-4,in an Enterobacter isolate from Scotland, abstr.1134010,”in *Program and Abstracts of the 15^th^ European Congress of Clinical Microbiology and Infectious Diseases*, Copenhagen.
27. Robledo IE, Moland ES, Aquino EA,Vazquez GJ, Sante MI, Bertran J, *et al.* (2007). “First report of a KPC-4 and CTX-M producing *K. pneumoniae* (KP) isolated from Puerto Rico, abstr. C2- 1933,”in *47th Annual Interscience Conference on Antimicrobial Agents and Chemotherapy* (Washington DC, American Society for Microbiology).
28. Wolter DJ, Kurpiel PM, Woodford N, Palepou MF, Goering RV. & Hanson ND. (2009). Phenotypic and enzymatic comparative analysis of the novel KPC variant KPC-5 and its evolutionary variants, KPC- 2 and KPC-4. *Antimicrob.Agents Chemother;* 53:557–562
29. Bartual SG, Seifert H, Hippler C, Luzon MA, Wisplinghoff H. & Rodriguez-Valera F. (2005). Development of amultilocus sequence typing scheme for characterization of clinical isolates of *Acinetobacter baumannii*. *J. Clin.Microbiol;* 43:4382–4390.
30. Perez F, Endimiani A, Ray AJ, Decker BK, Wallace CJ, Hujer KM, *et al.* (2010a). Carbapenem resistant *Acinetobacter baumannii* and *Klebsiella pneumoniae* across a hospital system: impact of post acute care facilities on dissemination. *J. Antimicrob. Chemother;* 65:1807–1818.
31. Da Silva GJ, Quinteira S, Bertolo E, Sousa JC, Gallego L, Duarte A. & Peixe L. (2004). Long-term dissemination of an OXA-40 carbapenemase- producing *Acinetobacter baumannii* clone in the Iberian Peninsula. *J. Antimicrob. Chemother;* 54:255–258.
32. Grosso F, Quinteira S. & Peixe L. (2011). Understanding the dynamics of imipenem resistant *Acinetobacter baumannii* lineages within Portugal. *Clin. Microbiol. Infec;* 17:1275–1279.
33. Vourli S, Giakkoupi P, Miriagou V, Tzelepi E, Vatopoulos AC. & Tzouvelekis LS. (2004). Novel GES/IBC extended-spectrum _-lactamase variants with carbapenemase activity in clinical Enterobacteria. *FEMS Microbiol. Lett;* 234:209–213.
34. Wachino J-I, Doi Y, Yamane K, Shibata N, Yagi T, Kubota T. & Arakawa Y. (2004). Molecular characterization of a cephamycin-hydrolyzing and inhibitor-resistant class A _-lactamase, GES-4, possessing a single G170S substitution in the omega-loop. *Antimicrob. Agents Chemother;* 48:2905–2910.
35. Viau RA, Hujer AM, Marshall SH, Perez F, Hujer KM, Briceño DF, *et al.* (2012). “Silent” dissemination of *Klebsiella pneumoniae* isolates bearing K. *pneumoniae* carbapenemase in a long term care facility for children and young adults in North east Ohio. *Clin. Infect.Dis;* 54:1314–1321
36. Jeong, S.H, Bae IK, Kim D, Hong SG, Song JS, Lee JH, & Lee SH. (2005). First outbreak of *Klebsiella pneumoniae* clinical isolates producing GES-5 and SHV-12 extended spectrum Beta lactamases in Korea. *Antimicrob. Agents Chemother;* 49:4809–4810.
37. Moubareck C, BremontS, Conroy MC, Courvalin P. & Lambert T. (2009). GES-11, a novel integron associated GES variant in *Acinetobacter baumannii*. *Antimicrob. Agents Chemother;* 53:3579–3581.
38. Bogaerts P, Verroken A, Jans B, Denis O. & Glupczynski Y. (2010). Global spread of New Delhi metallo-b-lactamase. *Lancet Infect Dis;* 10:831–832.
39. Naas, T. *et al.* (1994). Cloning and sequence analysis of the gene for a carbapenem-hydrolyzing class A b-lactamase, Sme-1, from *Serratia marcescens. Antimicrob. Agents Chemother;* 38:1262–1270
40. Deshpande LM, Jones RN, Fritsche TR. & Sader HS. (2006a). Occurrence and characterization of carbapenemase producing Enterobacteriaceae: report from the SENTRY antimicrobial surveillance program (2000–2004). *Microb.Drug Resist.* 12, 223–230.
41. Poirel L, Pitout JD. & Nordmann P. (2007). Carbapenemases: molecular diversity and clinical consequences. *Future Microbiol;* 2:501–512.
42. Carre¨r A, *et al.* (2008). Spread of OXA-48-positive carbapenem-resistant *Klebsiella pneumoniae* isolates in Istanbul, Turkey. *Antimicrob. Agents Chemother*; 52:2950–2954.
43. Queenan AM, Shang W, Schreckenberger P, Lolans K, Bush K. & Quinn J. (2006). SME-3, a novel member of the *Serratia marcescens* SME family of carbapenemhydrolyzing beta-lactamases. *Antimicrob. Agents Chemother;* 50:3485–3487.
44. Nordmann P, Mariotte S, Naas T, Labia R. & Nicolas MH. (1993). Biochemical properties of a carbapenem hydrolyzing beta-lactamase from *Enterobactercloacae* and cloning of the gene into *Escherichiacoli*. *Antimicrob.Agents Chemother;* 37:939–946.
45. Rasmussen BA, Bush K, Keeney D, Yang Y, Hare R., O’Gara C. & Medeiros AA. (1996). Characterization of IMI-1 beta-lactamase, a class A carbapenemhydrolyzing enzyme from Enterobacter cloacae. *Antimicrob. Agents Chemother*; 40:2080–2086.
46. Aubron C, Poirel L, Ash RJ. & Nordmann P. (2005). Carbapenemase producing *Enterobacteriaceae*, U.S. Rivers*. Emerg. Infect. Dis*. 11:260–264.
47. Yu Y-S, Du X-X, Zhou Z-H, Chen Y-G. & Li L-J. (2006). First isolation of *bla*IMI-2 in an *Enterobacter cloacae* clinical isolate from China. *Antimicrob. Agents Chemothe;.* 50:1610–1611.
48. Henriques I, Moura A Alves A, Saavedra MJ. & Correia A. (2004). Molecular characterization of a carbapenem-hydrolyzing class A ß-lactamase, SFC-1, from *Serratia fonticola* UTAD54. *Antimicrob. Agents Chemother;* 48:2321–2324.
49. Girlich D, Poirel L. & Nordmann P. (2010).Novel Ambler Class A Carbapenem-Hydrolyzing Beta Lactamase from a *Pseudomonas fluorescens* Isolate from the Seine River, Paris, France. *Antimicrobial Agents and Chemotherapy;* 54:328–332. 0066-4804/10/$12.00 doi:10.1128/AAC.00961-09.
50. Osano E, Arakawa Y, Wacharotayankun R, Ohta M, Horii T, Ito H, Yoshimura F. & Kato N. (1994). Molecular characterization of an enterobacterial metallo beta-lactamase found in a clinical isolate of *Serratia marcescens* that shows imipenem resistance. *Antimicrob. Agents Chemother*; 38:71–78.
51. Riccio ML, Franceschini N, Boschi L, Caravelli B, Cornaglia G, Fontana R, Amicosante G. & Rossolini GM. (2000). Characterization of the metallo-_-lactamase determinant of *Acinetobacter baumannii* AC-54/97 reveals the existence of *bla*IMP allelic variants carried by gene cassettes of different phylogeny. *Antimicrob. Agents Chemother;* 44:1229–1235.
52. Lauretti L, *et al.* (1999). Cloning and characterization of blaVIM, a new integron-borne metallo-beta-lactamase gene from a Pseudomonas aeruginosa clinical isolate. *Antimicrob. Agents Chemother;* 43:1584 –1590.
53. Poirel L, Le Thomas I, Naas T, Karim A. & Nordmann, P. (2000b). Biochemical sequence analyses of GES-1, a novel class A extended-spectrum beta-lactamase, and the class 1 integron In52 from Klebsiella pneumoniae. *Antimicrob. Agents Chemother;* 44:622–632
54. Yong D, Toleman MA, Giske CG, Cho HS, Sundman K, Lee K.& Walsh TR. (2009). Characterization of a new metallo-beta-lactamase gene, blaNDM-1, and a novel erythromycin esterase gene carried on a unique genetic structure in Klebsiella pneumoniae sequence type 14 from India. *Antimicrob Agents Chemother*; 53:5046–5054.
55. Kaase M, Nordmann P, Wichelhaus TA, Gatermann SG, Bonnin RA. & Poirel L. (2011). NDM-2 carbapenemase in *Acinetobacter baumannii* from Egypt. *J Antimicrob Chemother*; 66;1260–1262.
56. Nordmann P, Girlich D and Poirel L, 2012a. Detection of Carbapenemase Producers in Enterobacteriaceae by Use of a Novel Screening Medium. Journal of clinical microbiology,50, 2761-2766.
57. Lee, K., J. H. Yum, D. Yong, H. M. Lee, H. D. Kim, J. D. Docquier, G. M. Rossolini, and Y. Chong. 2005. Novel acquired metallo-_-lactamase gene, *bla*SIM-1, in a class 1 integron from *Acinetobacter baumannii* clinical isolates from Korea. *Antimicrob. Agents Chemother*; 49:4485–4491.
58. Afzal-Shah M, Woodford N & Livermore DM. (2001). Characterization of OXA-25, OXA-26, and OXA-27, molecular class D Beta lactamases associated with carbapenem resistance in clinical isolates
59. Gogou V, Pournaras S, Giannouli M, Voulgari E, Piperaki E-T, Zarrilli R, et al. (2011). Evolution of multidrug-resistant *Acinetobacter baumannii* clonal lineages: a 10year study in Greece (2000–09). *J. Antimi- crob.Chemother;* 66:2767–2772.
60. Bou G, Oliver A. & Martinez-Beltran J. (2000). OXA-24, a novel class D _-lactamase with carbapenemase activity in an *Acinetobacter baumannii* clinical strain. *Antimicrob. Agents Chemother*; 44:1556–1561.
61. Lopez-Otsoa F, Gallego L, Towner KJ, Tysall L, Woodford N. & Livermore DM. (2002). Endemic carbapenem resistance associated with OXA-40 carbapenemase among *Acinetobacter baumannii* isolates from a hospital in northern Spain. *J. Clin. Microbiol.* 40:4741–4743.
62. Poirel L, He´ritier C, Tolu¨n V. & Nordmann P. **(**2004). Emergence of oxacillinase-mediated resistance to imipenem in *Klebsiella pneumoniae*. *Antimicrob. Agents Chemother;* 48**:**15–22.
63. Brown S, Young HK. & Amyes SG. (2005). Characterisation of OXA-51, a novel class D carbapenemase found in genetically unrelated clinical strains of *Acinetobacter baumannii* from Argentina. Clin. Microbiol. Infect; 11:15–23.
64. Evans BA, Brown S, Hamouda A, Findlay J. & Amyes SGB. (2007). Eleven novel OXA-51-like enzymes from clinical isolates of *Acinetobacter baumannii*. *Clin.Microbiol.Infect;* 13:1137–1138.
65. Poirel L, Marque´ S, He´ritier C, Segonds C, Chabanon G. & Nordmann P. (2005). OXA-58, a novel class D Beta lactamase involved in resistance to carbapenems in *Acinetobacter baumannii*. *Antimicrob. Agents Chemother*; 49:202–208.
66. Higgins PG, Poirel L, Lehmann M, Nordmann P. & Seifert H. (2009). OXA-143, a novel carbapenem hydrolyzing classD beta-lactamase in *Acinetobacter baumannii*. *Antimicrob. AgentsChemother;* 53:5035–5038.
67. Poirel L, Castanheira M, Carrer A, Rodriguez CP, Jones RN, Smayevsky J. & Nordmann P. (2011b). OXA-163, an OXA-48-Related Class D Beta Lactamase with Extended Activity Toward Expanded-Spectrum Cephalosporins. Antimicrobial Agents and chemotherapy, June 2011, p. 2546–2551 Vol. 55, No. 6 0066-4804/11/$12.00 doi:10.1128/AAC.00022-11
68. Abdelaziz MO, Bonura C, Aleo A, El-Domany RA, Fasciana T. & Mammina C. (2012). OXA-163- producing *Klebsiella pneumoniae* in Cairo, Egypt, in 2009 and 2010. *J. Clin. Microbiol;* 50:2489–2491.
69. Castanheira M, Deshpande LM, Mathai D, Bell JM, Jones RN. & Mendes RE. (2011). Early dissemination of NDM-1- and OXA- 181-producing Enterobacteriaceae in Indian hospitals: report from the SENTRY Antimicrobial Surveillance Program, 2006–2007. *Antimicrob Agents Chemother*; 55:1274–1278.
70. Potron A, Nordmann P. & Poirel L. (2013). Characterizationof OXA-204, acarbapenem-hydrolyzing class D β-lactamase from *Klebsiella pneumoniae*. *Antimicrob.Agents Chemother;* 57:633–636.
71. Poirel L, Potron A. & Nordmann P. (2012c). OXA-48-like carbapenemases: the phantom menace. *J. Antimicrob.Chemother;* 67:1597– 1606.
72. Robledo IE, Vázquez GJ, Aquino EA, Moland ES, Santé MI. & Hanson ND. (2008). A novel KPC variant, KPC-6 in a *Klebsiella pneumoniae* isolated in Puerto Rico, abstr.C2-3738,” in 48^th^ Annual Interscience Conference on Antimicrobial Agents and Chemotherapy and the 46^th^ Annual Meeting of Infectious Diseases Society of America Washington DC. *American Society for Microbiology.*
73. Kitchel B, Rasheed JK, Patel JB, Srinivasan A, Navon-Venezia S. & Carmeli Y. (2009a). Molecular epidemiology of KPC-producing *Klebsiella pneumoniae* isolates in the United States: clonal expansion of multilocus sequence type258. *Antimicrob Agents Chemother;* 53: 3365–3370.
74. Navon-Venezia S, Leavitt A, Schwaber MJ, Rasheed JK, Srinivasan A and Patel JB. (2009). First report on a hyper epidemic clone of KPC-3-producing *Klebsiella pneumoniae* in Israel genetically related to a strain causing outbreaks in the United States. *Antimicrob.Agents Chemother;* 53:818–820
75. Gregory CJ, Lata E, Stine N, Gould C, Santiago LM. & Vazquez GJ. (2010). Outbreak of carbapenemresistant *Klebsiella pneumoniae* in Puerto Rico associated with a novel carbapenemase variant. *Infect.Co*
76. Nordmann P, Dortet L. & Poirel L. (2012). Carbapenem resistance in Enterobacteriaceae: here is the storm! *Trends in Molecular Medicine* Vol. 18, No. 5
77. UNAS. (2015). Antibiotic Resistance in Uganda: Situation Analysis and Recommendations
78. Kateete DP, Nakanjako R, Namugenyi J, Erume J, Joloba ML. & Najjuka CF. (2016). Carbapenem resistant Pseudomonas aeruginosa and Acinetobacter baumannii at Mulago Hospital in Kampala, Uganda (2007–2009) *Springer Plus* 5:1308 DOI 10.1186/s40064-016-2986-7
79. Okoche D, Asiimwe BB, Katabazi FA, Kato L. & Najjuka CF. (2015). Prevalence and Characterization of Carbapenem-Resistant Enterobacteriaceae Isolated from Mulago National Referral Hospital, Uganda. *PLOS ONE* | DOI:10.1371/journal.pone.0135745
80. Mushi MF, Mshana SE, Imirzalioglu C and Bwanga F. (2014) Carbapenemase genes among multidrugresistant gram negative clinical isolates from a tertiary hospital in Mwanza, Tanzania*. Biomed Res Int*.
81. Poirel L, Revathi G, Bernabeu S, & Nordman P. (2011). Detection of NDM-1-Producing Klebsiella pneumonia in Kenya. American Society for Microbiology, *Antimicrobial Agents and Chemotherapy;* 55: 934-936
82. Crowley B, Bened VJ, & Dome´nech-Sa´ nchez A. (2002). Expression of SHV-2 b-lactamase and of reduced amounts of OmpK36 porin in Klebsiella pneumoniae results in increased resistance to cephalosporins and carbapenems. *Antimicrob Agents Chemother*; 46: 3679–3682.
83. Yang, D., Guo, Y. & Zhang, Z. (2009). Combined porin loss and extended spectrum beta lactamase production is associated with an increasing imipenem minimal inhibitory concentration in clinical Klebsiella pneumoniae strains. *Curr Microbiol;* 58:366–370.
